# The role of laparoscopy in emergency colorectal surgery

**DOI:** 10.15537/smj.2022.43.12.20220658

**Published:** 2022-12

**Authors:** Nahar A. Alselaim, Haifa A. Altoub, Mohammed K. Alhassan, Raghad M. Alhussain, Abdullah A. Alsubaie, Farah A. Almomen, Abrar M. Almutairi, Sultanah F. Bin Gheshayan

**Affiliations:** *From College of Medicine (Alselaim, Altoub, Bin Gheshayan), King Saud bin Abdulaziz University for Health Sciences; from the Research Unit (Almutairi), College of Applied Medical Science, King Saud bin Abdulaziz University for Health Sciences; from the College of Medicine (Alhassan), King Saud University Medical City; from the Department of Surgery (Alselaim, Almutairi, Bin Gheshayan), King Abdullah International Medical Research Center; from the Department of General Surgery (Alselaim, Bin Gheshayan), Ministry of National Guard- Health Affairs; from College of Medicine (Almomen), Imam Mohammad Ibn Saud Islamic University, Riyadh; and from College of Medicine, King Khalid University, Abha, Kingdom of Saudi Arabia (Alhussain, Alsubaie).*

**Keywords:** laparoscopic, emergency, colorectal, surgery

## Abstract

**Objectives:**

To assess the outcomes of the laparoscopic approach compared to those of the open approach in emergency colorectal surgery.

**Methods:**

This retrospective cohort study included all patients aged >15 years who underwent emergency colorectal surgery from 2016-2021 at King Abdulaziz Medical City, Riyadh, Saudi Arabia. Patients were divided based on the surgical approach into laparoscopic and open groups.

**Results:**

A total of 241 patients (182 open resections, 59 laparoscopic approaches) were included in this study. The length of stay in the intensive care unit was shorter in the laparoscopic than in the open group (1±3 days vs. 7±16 days). After multivariable logistic regression, patients undergoing laparoscopic resection had a 70% lower risk of surgical site infection than those undergoing open surgery (adjusted odds ratio=0.33, 95% confidence interval: [0.06-1.67]), a difference that was not significant (*p*=0.18). Lastly, patients who underwent open surgery had a high proportion of 30-day mortality (n=26; 14.3%), compared to those who underwent laparoscopic resection (n=2; 3.4%, *p*=0.023).

**Conclusion:**

Laparoscopy in emergency colorectal surgery is safe and feasible, with a trend toward better outcomes. Colorectal surgery specialization is an independent predictor of an increased likelihood of undergoing laparoscopy in emergency colorectal surgery.

**C**olorectal cancer (CRC) is the third most commonly diagnosed malignancy worldwide, with a rising incidence. In 2020, more than 1.9 million new cases of CRC were diagnosed.^1^ A recent study demonstrated that 33% of patients with CRC required emergent surgical intervention.^2^

There has been a continuous increase in the use of the laparoscopic approach for elective colorectal surgeries, with evidence of better surgical and patient-reported outcomes, including fewer complications, earlier return of gastrointestinal (GI) function, less postoperative pain, and shorter length of hospital stay (LOS) compared with those after open surgery.^3-6^

Introduction of the laparoscopic approach has revolutionized the field of minimally invasive surgery, and it has been widely adopted in many specialties. However, despite its widespread use in elective surgery, it is unclear whether this technique can be used in emergency colorectal settings.^7,8^ Most global studies on this topic have been context-specific with the range of presenting pathology, with the strongest evidence for procedures such as appendicitis, cholecystitis, diverticular disease, and malignancies.^9-14^

However, few studies have addressed the safety and feasibility of laparoscopic colorectal surgery in emergency settings; therefore, this study aimed to assess the outcomes of laparoscopic colon surgery in terms of mortality and morbidity compared with those of open surgery in emergency settings.

## Methods

This retrospective cohort study included 241 patients who underwent emergency laparoscopic and open colorectal surgery from July 2016 to July 2021 at King Abdulaziz Medical City, Riyadh, Saudi Arabia. Patients who were less than 15 years old, underwent other major surgical procedures at the same time, or underwent elective colorectal surgery were excluded.

A chart review technique was used, using the BestCare system, to collect patient characteristics (age, gender, body mass index [BMI], urgency, surgeon specialty, diagnosis, American Society of Anesthesiologists [ASA] classification, white blood cell count [WBC], preop-sepsis, smoking, anticoagulation, steroid, and comorbidities), hospital characteristics (amount of blood loss, stoma, and type of resection), and postoperative outcomes (LOS, 30-day mortality [30D], intensive care unit-LOS [ICU-LOS], surgical site infection [SSI], readmission, reoperation, and complications) among patients who underwent laparoscopic surgery. The data were entered and coded in Microsoft Excel and then imported to Statistical Package for the Social Sciences, version 25.0 (IBM Corp., Armonk, NY, USA) software. This study was carried out according to the guidelines of the Declaration of Helsinki. It was approved by the Institutional Review Board (ethics approval number: RSS21R/020/07).

### Statistical analysis

Statistical analyses were carried out using Statistical Package for the Social Sciences, version 25.0 (IBM Corp., Armonk, NY, USA). Categorical variables are presented as proportions and continuous variables as mean ± standard deviation (SD). Pearson χ^2^ test was used for categorical variables and independent t-test was used for continuous variables to investigate the differences between subjects who underwent laparoscopic and open surgery in terms of patient and hospital characteristics. Binary logistic regression (univariate and multivariate) was used to estimate the odds ratio (OR) of undergoing laparoscopic resection to adjust for patient characteristics (age, gender, BMI, urgency, surgeon specialty, diagnosis, ASA class, WBC, preop-sepsis, smoker, anticoagulation, steroid, and comorbidities) and hospital characteristics (amount of blood loss, stoma, and type of resection). Linear and binary logistic regressions were carried out to estimate the postoperative outcomes (LOS, 30D mortality, ICU-LOS, SSI, readmission, reoperation, and complications) among patients who underwent laparoscopic surgery, with adjustments for the patient and hospital characteristics. A *p*-value of <0.05 and 95% confidence intervals (CI) were used to report the statistical significance and precision of results.

## Results

The baseline characteristic distributions presented in Table 1 include patient, surgeon, and hospital characteristics of patients who underwent open resection and laparoscopic resection. Of the 241 resections, 182 were open resections and 59 laparoscopic resections. Most patients were in the age group of 50-64 years, with 30.2% undergoing open resection and 42.5% undergoing laparoscopic resection. Most participants were male (n=104), and there was no significant difference in the gender distribution between patients who underwent open resection and those who underwent laparoscopy (*p*=0.092; Table 1). A total of 150 patients underwent urgent open surgery, and 41 patients underwent urgent laparoscopic resection; there was a statistical difference between the 2 groups (*p*=0.033).

**Table 1:** - Patient’s characteristics according to surgical approach (N=241).

Variables	Open resection	Laparoscopic	*P*-values
***Age***			
18-49	51 (28.1)	11 (18.6)	0.279
50-64	55 (30.2)	25 (42.5)	
65-74	35 (19.2)	12 (20.3)	
>75	41 (22.5)	11 (18.6)	
***Gender***			
Male	104 (57.1)	41 (69.5)	0.092
Female	78 (42.9)	18 (30.5)	
***Body mass index***			
18.5-24.9	67 (37.4)	26 (44.1)	0.761
25.0-29.9	56 (31.3)	15 (25.4)	
30.0-34.9	37 (20.7)	11 (18.6)	
≥35	19 (10.6)	7 (11.9)	
**Diagnosis**			
Malignance	99 (54.4)	39 (66.1)	0.114
Non-malignance	83 (45.6)	20 (33.9)	
***ASA class***			
ASA class 1 (0, 1, and 2)	64 (35.4)	20 (33.9)	0.838
ASA class 2 (3, 4, and 5)	117 (64.6)	39 (66.1)	
***Anticoagulation***			
Yes	37 (20.4)	18 (30.5)	0.110
No	144 (79.6)	41 (69.5)	
***Procedure***			
Right hemicolectomy	50 (27.4)	12 (20.3)	0.010
Left hemicolectomy	56 (30.5)	13 (25.5)	
Subtotal and total proctocolectomy	13 (7.0)	3 (5.0)	
Hartmann-procedure	28 (14.5)	5 (7.0)	
Stoma formation	33 (19.1)	25 (41.0)	
Others	2 (1.5)	1 (1.2)	
***Steroid***			
Yes	8 (4.4)	4 (6.8)	0.470
No	173 (95.6)	55 (93.2)	
***Non-colorectal surgeon***			
Yes	101 (55.5)	43 (72.9)	0.018
No	81 (44.5)	16 (27.1)	
***Type of resection***			
Total	8 (4.4)	5 (8.5)	0.000
Segmental	141 (77.5)	29 (49.2)	
Stoma	33 (18.1)	25 (42.4)	
***Urgency***			
<48 hours	150 (82.4)	41 (69.5)	0.033
>48 hours	32 (17.6)	18 (30.5)	
WBC, mean±SD	11.75±7.18	10.11±9.13	0.439
***Pre-op sepsis***			
Yes	58 (31.9)	5 (9.4)	0.001
No	124 (68.1)	48 (90.6)	
***Smoker***			
Yes	23 (12.6)	7 (13.0)	0.950
No	159 (87.4)	47 (87.0)	

Values are presented as a number and precentage (%). ASA: American Society of Anesthesiologists, WBC: white blood cell, BL: Burkitt’s lymphoma, SD: standard deviation

**Table 1:** - Patient’s characteristics according to surgical approach (N=241). (Continuation)

Variables	Open resection	Laparoscopic	*P*-values
***Co-morbid***			
***Pulmonary***			
Yes	40 (22.0)	6 (10.2)	0.045
No	142 (78.0)	53 (89.8)	
***Cardiac***			
Yes	100 (54.9)	42 (71.2)	0.028
No	82 (45.1)	17 (28.8)	
***Endocrine***			
Yes	94 (51.6)	26 (44.1)	0.312
No	88 (48.4)	33 (55.9)	
***Hepatic***			
Yes	14 (7.7)	5 (8.5)	0.846
No	168 (92.3)	54 (91.5)	
***Renal***			
Yes	32 (17.6)	10 (16.9)	0.911
No	150 (82.4)	49 (83.1)	
***Hematology***			
Yes	19 (10.4)	11 (18.6)	0.097
No	163 (89.6)	48 (81.4)	
***Neurology***			
Yes	26 (14.3)	10 (16.9)	0.618
No	156 (85.7)	49 (83.1)	
***No co-morbid***			
Yes	18 (9.9)	2 (3.4)	0.116
No	164 (90.1)	57 (96.6)	
***Other co-morbid***			
Yes	15 (8.2)	1 (1.7)	0.079
No	167 (91.8)	58 (98.3)	
Amount BL, mean±SD	182.9±283.4	75.59±124.9	0.003
***Stoma***			
Yes	143 (78.6)	38 (64.4)	0.029
No	39 (21.4)	21 (35.6)	

Values are presented as a number and precentage (%). ASA: American Society of Anesthesiologists, WBC: white blood cell, BL: Burkitt’s lymphoma, SD: standard deviation

In terms of perioperative outcomes, there was a significant difference between the 2 groups of 63 patients who presented with a preoperative sepsis outcome in the open resection (31.9%) and laparoscopic resection (9.4%) groups. Regarding the type of procedure, stoma formation was more common in patients who underwent laparoscopy (41%), while the left hemicolectomy procedure was more common in patients who underwent open surgery (30%; *p*=0.010). Pulmonary comorbidities were more common in the open surgery group (*p*=0.045), while cardiac comorbidities were more common in the laparoscopic surgery group (*p*=0.028). Mean blood loss was significantly different between the 2 groups (*p*=0.003). However, there was no significant difference in the mean WBC between the 2 groups (*p*=0.439; Table 1).

As shown in Table 2, multivariable logistic regression was carried out to adjust for the correlation between patient and hospital characteristics. After adjustment, patients in the age group of 18-49 years had a 24% (95% CI: [0.02-2.43]) decrease in the odds of having laparoscopic resection, which was not statistically significant (*p*=0.22). Furthermore, obese people had a 52% (95% CI: [0.05-5.15]) lower chance of undergoing laparoscopic surgery than overweight people, and the result was not significant (*p*=0.58). Patients who underwent laparoscopic resection were 4.7 (95% CI: [1.10-19.7]) times more likely to have anticoagulation therapy, and the result was statistically significant (*p*=0.03). Patients with pulmonary and cardiac comorbidities had a higher chance of undergoing laparoscopic resections than patients with other comorbidities, such as endocrine (20%), hepatic (80%), and renal diseases (60%). The results were not statistically significant. On the other hand, patients operated on by colorectal surgeons had an 83% (95% CI: [0.04-0.71]) increased chance of undergoing a laparoscopic resection compared to patients operated on by non-colorectal surgeons. This result was statistically significant (*p*=0.01; Table 2).

**Table:** Table 2 - Adjusted odds ratio of undergoing laparoscopic approach in emergency settings.

Variables	Adjusted estimate	*P*-values
***Age***		
18-49	0.24 (0.02-2.43)	0.22
50-65	0.56 (0.03-8.83)	0.68
65-74	0.40 (0.03-4.71)	0.47
***Gender***		
Male	2.23 (0.52-9.48)	0.27
Female	Ref	
***Body mass index***		
18.5-24.9	0.34 (0.06-1.94)	0.23 0.91 0.58
25.0-29.9	0.89 (0.12-6.55)	
30.0-34.9	0.52 (0.05-5.15)	
≥35	Ref	
***Diagnosis***		
Malignance	0.67 (0.15-2.83)	0.58
Non-malignance	Ref	
***ASA class***		
ASA class 1 (0, 1, and 2)	0.22 (0.02-2.0)	0.18
ASA class 2 (3, 4, and 5)	Ref	
***Anticoagulation***		
Yes	4.67 (1.10-19.7)	0.03
No	Ref	
***Steroid***		
Yes	2.10 (0.17-24.8)	0.55
No	Ref	
***Non-colorectal surgeon***		
Yes	0.17 (0.04-0.71)	0.01
No	Ref	Ref
***Type of resection***		
Total	6.13 (0.30-123.1)	0.23 0.32
Segmental	5.22 (0.19-137.2)	
Stoma	Ref	
***Urgency***		
<48 hours	1.35 (0.14-12.3)	0.78
>48 hours	Ref	

Values are presented as an odd ratio and 95% confidence interval (CI). ASA: American Society of Anesthesiologists, WBC: white blood cell, BL: Burkitt’s lymphoma

**Table 2:** - Adjusted odds ratio of undergoing laparoscopic approach in emergency settings. (Continuation)

Variables	Adjusted estimate	*P*-values
WBC	1.01 (0.93-1.09)	0.77
***Pre-op sepsis***		
Yes	2.71 (0.63-11.5)	0.17
No	Ref	
***Smoker***		
Yes	0.39 (0.04-3.78)	0.41
No	Ref	
***Co-morbid***		
***Pulmonary***		
Yes	1.78 (0.42-7.49)	0.43
No	Ref	
***Cardiac***		
Yes	1.22 (0.20-7.15)	0.82
No	Ref	
***Endocrine***		
Yes	0.82 (0.17-3.89)	0.81
No	Ref	
***Hepatic***		
Yes	0.22 (0.03-1.46)	0.12
No	Ref	
***Renal***		
Yes	0.44 (0.09-2.04)	0.29
No	Ref	
***Hematology***		
Yes	2.06 (0.35-11.9)	0.41
No	Ref	
***Neurology***		
Yes	6.80 (0.88-52.2)	0.065
No	Ref	
***No co-morbid***		
Yes	000	0.998
No	Ref	
***Other co-morbid***		
Yes	1.01 (0.09-10.7)	0.993
No	Ref	
Amount BL	0.99 (0.996-1.0)	0.06
***Stoma***		
Yes	3.62 (0.31-41.4)	0.30
No	Ref	

Values are presented as an odd ratio and 95% confidence interval (CI). ASA: American Society of Anesthesiologists, WBC: white blood cell, BL: Burkitt’s lymphoma

The mean LOS for patients undergoing laparoscopic resection was 14±18 days and for those undergoing open surgery was 23±28 days (*p*=0.005; Table 3). However, patients who underwent open surgery had a high proportion of 30D mortality (n=26; 14.3%), compared to those who underwent laparoscopic resection (n=2; 3.4%). The patients who had laparoscopic resection had 85% lower odds of 30D mortality than patients who had open surgery (adjusted OR=0.15, 95% CI: [0.01-1.8]), and this difference was not statistically significant (*p*=0.13). Further, only 7 patients with laparoscopic resection had a surgical site infection. After adjustment, patients undergoing laparoscopic resection had a 70% lower risk of surgical site infection than those undergoing open surgery (adjusted OR=0.33, 95% CI: [0.06-1.67]), a difference that was also not significant (*p*=0.18).

**Table 3:** - Distribution of mortality and morbidity by type of intervention.

Postoperative outcomes	Open resection	Laparoscopic	*P*-values
Length of stay, mean±SD	23.52±28.8	14.15±18.1	0.005
Intensive care unit length of stay, mean±SD	7.27±16.1	1.24±3.08	0.000
***30 days mortality***			
Yes	26 (14.3)	2 (3.4)	0.023
No	156 (85.7)	57 (96.6)	
***Surgical site infection***			
Yes	37 (20.4)	7 (13.0)	0.216
No	144 (79.6)	47 (87.0)	
***Readmission***			
Yes	12 (6.6)	10 (16.9)	0.016
No	170 (93.4)	49 (83.1)	
***Reoperation***			
Yes	37 (20.3)	7 (11.9)	0.144
No	145 (79.7)	52 (88.1)	
***Complications***			
***Septic shock***			
Yes	15 (8.2)	3 (5.1)	0.423
No	167 (91.8)	56 (94.9)	
***Cardiovascular***			
Yes	7 (3.8)	3 (5.1)	0.678
No	175 (96.2)	56 (94.9)	
***Pulmonary***			
Yes	9 (4.9)	2 (3.4)	0.619
No	173 (95.1)	57 (96.6)	
***GI***			
Yes	14 (7.7)	3 (5.1)	0.497
No	168 (92.3)	56 (94.9)	
***GU***			
Yes	16 (8.8)	2 (3.4)	0.170
No	166 (91.2)	57 (96.6)	
***Endocrine***			
Yes	0 (0.0)	1 (1.7)	0.078
No	182 (100)	58 (98.3)	
***Peritonitis***			
Yes	3 (1.6)	1 (1.7)	0.981
No	179 (98.4)	58 (98.3)	
***Neurology***			
Yes	2 (1.1)	1 (1.7)	0.720
No	180 (98.9)	58 (98.3)	

Values are presented as a number and percentage (%). GI: *gastrointestinal,* GU: *genitourinary,* SD: standard deviation

The proportion of readmissions was slightly greater in patients who underwent laparoscopic resection (16.9% vs. 6.6%), whereas the proportion of reoperations was lower in patients who underwent laparoscopic resection (11.9% vs. 20.3%). Patients who underwent laparoscopic resection were 2.31 times more likely to have a readmission than those who underwent open surgery (adjusted OR=2.31, 95% CI: [0.52-10.23]). However, these differences were no longer significant in the multi-regression model. Concerning complications, patients who underwent laparoscopic surgery had slightly lower rates of complications (5.1% vs. 8.2%) than those who underwent open surgery (5.1% vs. 7.7%). With this adjustment, patients who had laparoscopic surgery had decreased odds of complications such as septic shock (OR=0.36, 95% CI: [0.04-2.92]), GI (OR=0.23, 95% CI: [0.004-14.6]), and genitourinary (GU) (OR=0.03, 95% CI: [0.001-1.16]) compared to patients who had open surgery. However, there was no significant association between the complications and laparoscopic resection (p=0.33, p=0.36, and p=0.49) (Table 4).

**Table 4:** - Adjusted outcomes of laparoscopic versus open resection.

Postoperative outcomes	Adjusted estimate	*P*-values
Mean difference (B) length of stay	-4.62 (-13.4-4.23)	0.30
Mean difference (B) intensive care unit length of stay	-3.12 (-7.39-1.15)	0.15
30 days mortality	0.15 (0.01-1.8)	0.13
Surgical site infection	0.33 (0.06-1.67)	0.18
Readmission	2.31 (0.52-10.23)	0.26
Reoperation	0.37 (0.11-1.29)	0.12
***Complications***		
Septic shock	0.36 (0.04-2.92)	0.33
Cardiovascular	00 (0.0-1.0)	0.25
Pulmonary	0.007 (0.00-293.5)	0.36
GI	0.23 (0.004-14.6)	0.49
GU	0.03 (0.001-1.16)	0.06

Values are presented as odds ratio and 95% confidence interval (CI). GI: gastrointestinal, GU: genitourinary

## Discussion

This study aimed to assess the outcomes of laparoscopic colorectal surgery in terms of mortality and morbidity compared with those of open surgery in emergency settings.

Emergency colorectal surgery comprises a heterogeneous set of patients with different diagnoses and physical statuses. Traditional practice has always advocated for an open approach, especially in ill patients, to avoid longer operative time and pneumoperitoneum during laparoscopy, both of which might affect the hemodynamics of the patients and subsequently their postoperative outcomes.^15^

After multivariate regression analysis, we found no differences in the postoperative outcomes between the laparoscopic and open approaches. Several studies have attempted to address the role of laparoscopy in emergency colorectal surgery with controversial results. Most of these studies have demonstrated that laparoscopy is equivalent to open surgery in emergency settings, with some demonstrating better outcomes with laparoscopy. A population-based study carried out in England showed that there was a statistical difference in the median LOS and lower 90-day mortality. However, patient characteristics were not fully adjusted for all differences.^16^ Another population-based study carried out in the United States found a statistically significant reduction in LOS, mortality rate, and all complication rates in the laparoscopic group.^17^ Moreover, a recent multicenter feasibility randomized clinical trial was carried out with 64 patients who showed an acceptable safety profile for laparoscopy in emergency colorectal surgery.^18^

One of the several advantages of laparoscopy in elective colorectal surgery is less blood loss than in the open approach.^19^ Our results demonstrated that, in emergency settings, the laparoscopic approach had a statistically significant lower mean blood loss than open colorectal surgery (*p*=0.003).

One of the controversial factors in using the laparoscopic approach in emergency settings is the physical status of the patient, which is measured using the ASA score. According to a recent study, patients with poor ASA scores had a lower chance of undergoing laparoscopy.^16^ However, another study found that laparoscopy was safe in selected patients with ASA scores of <3 (patients with a score of 4 were not studied).^20^ This study builds on the previous one by adding on the safety of this approach to all ASA scores, as the laparoscopic approach was used in 39 patients (66.1% in laparoscopic group); an ASA score of ≥3 was found to be safe in terms of amount of blood loss, 30D mortality, complications, LOS, ICU-LOS, superficial skin infection, and reoperation.

Although not statistically significant, almost half of our patients presenting to the emergency department were diagnosed with malignancies, with 99 patients who underwent the open approach and 39 who underwent laparoscopic surgery. Most of our patients were in the age group of 50-64 years, with 30.2% undergoing open resection and 42.5% undergoing laparoscopic resection. Moreover, among patients aged ≥65 years, 76 (41.7%) underwent the open approach and 23 (38.9%) the laparoscopic approach. This is consistent with the findings of a recent study showing that the emergency presentation of CRC is more common in elderly patients.^21^

Similar to a large study by Keller et al,^17^ colorectal surgeons were the only significant variable that predicted the increased likelihood of a patient undergoing the laparoscopic approach in emergency settings. Patients operated on by colorectal surgeons had an 83% increased chance of undergoing a laparoscopic approach compared to patients operated on by non-colorectal surgeons. Another propensity score-matched study showed that 88.9% of emergency laparoscopic colorectal colectomies were carried out by colorectal surgeons.^22^ This observation aligns with several studies showing that colorectal surgery specialization is an independent factor for better outcomes in patients undergoing colorectal surgery.^23^

This study showed that the laparoscopic approach in emergency settings is safe and feasible, with a trend towards better postoperative outcomes in line with growing evidence in the literature regarding the role of laparoscopy in emergency colorectal surgery.

### Study limitations

Its retrospective nature, may have impacted our results. In addition, the sample size might hinder the detection of significant associations when the adjustment of variables is attempted. Therefore, to demonstrate the role of laparoscopy in emergency colorectal surgery, future studies with larger randomized clinical trials are needed.

In conclusion, the use of laparoscopy in emergency colorectal surgery is safe and feasible, with a trend toward better outcomes. Colorectal surgery specialization is an independent predictor of an increased likelihood of undergoing laparoscopy in emergency colorectal surgery.
